# Association of the solute carrier family 11 member 1 gene polymorphisms with susceptibility to leprosy in a Brazilian sample

**DOI:** 10.1590/0074-02760150326

**Published:** 2016-02

**Authors:** Maria José Franco Brochado, Maria Fernanda Chociay Gatti, Marco Antônio Zago, Ana Maria Roselino

**Affiliations:** 1 Universidade de São Paulo, Faculdade de Medicina de Ribeirão Preto, Hospital Universitário, Departamento de Clínica Médica, Divisão de Dermatologia, Ribeirão Preto, SP, Brasil; 2 Universidade de São Paulo, Faculdade de Medicina de Ribeirão Preto, Hospital Universitário, Departamento de Clínica Médica, Divisão de Hematologia, Ribeirão Preto, SP, Brasil; 3 Universidade de São Paulo, Faculdade de Medicina de Ribeirão Preto, Centro de Terapia Celular, Ribeirão Preto, SP, Brasil

**Keywords:** Slc11a1/Nramp1, leprosy, polymorphism

## Abstract

Natural resistance-associated macrophage protein 1/solute carrier family 11 member 1 gene (*Nramp1/Slc11a1*) is a gene that controls the susceptibility of inbred mice to intracellular pathogens. Polymorphisms in the human *Slc11a1/Nramp1* gene have been associated with host susceptibility to leprosy. This study has evaluated nine polymorphisms of the *Slc11a1/Nramp1* gene [(GT)n, 274C/T, 469+14G/C, 577-18G/A, 823C/T, 1029 C/T, 1465-85G/A, 1703G/A, and 1729+55del4] in 86 leprosy patients (67 and 19 patients had the multibacillary and the paucibacillary clinical forms of the disease, respectively), and 239 healthy controls matched by age, gender, and ethnicity. The frequency of allele 2 of the (GT)n polymorphism was higher in leprosy patients [p = 0.04, odds ratio (OR) = 1.49], whereas the frequency of allele 3 was higher in the control group (p = 0.03; OR = 0.66). Patients carrying the 274T allele (p *=* 0.04; OR = 1.49) and TT homozygosis (p = 0.02; OR = 2.46), such as the 469+14C allele (p = 0.03; OR = 1.53) of the 274C/T and 469+14G/C polymorphisms, respectively, were more frequent in the leprosy group. The leprosy and control groups had similar frequency of the 577-18G/A, 823C/T, 1029C/T, 1465-85G/A, 1703G/A, and 1729+55del4 polymorphisms. The 274C/T polymorphism in exon 3 and the 469+14G/C polymorphism in intron 4 were associated with susceptibility to leprosy, while the allele 2 and 3 of the (GT)n polymorphism in the promoter region were associated with susceptibility and protection to leprosy, respectively.

Leprosy is a chronic granulomatous infection affecting the skin and peripheral nerves; it is caused by the obligate intracellular bacillus *Mycobacterium leprae*(Walker & Lockwood 2007). The disease is characterised by a spectrum of clinical manifestations - from the tuberculoid (TT) to the lepromatous (LL) poles - which correlate with the host cell-mediated immunity against the bacillus. In patients with TT leprosy or the paucibacillary (PB) clinical form of the disease, the *M. leprae*-specific T-helper (Th)1-type immunity is robust, which limits the clinical disease. Only a few bacilli appear in skin biopsy lesions. LL leprosy or the multibacillary (MB) clinical form of the disease is characterised by low cell-mediated immunity with a humoral Th2 response. The patients display disseminated infection and high bacillary load in skin biopsy lesions. In-between these two polar forms, unstable borderline (BB) cases with specific clinical, immunological, and pathological characteristics exist (Ridley & Jopling 1966). Besides environmental and lifestyle factors, host genetic susceptibility has been attributed to the clinical diversity of the disease (Misch et al. 2010).

The solute carrier family 11 member 1 gene (*Slc11a1*), also known as natural resistance-associated macrophage protein 1 (*Nramp1*), is a gene that controls the susceptibility of inbred mice to intracellular pathogens, including*Mycobacterium*, *Salmonella*, and*Leishmania* microorganisms (Vidal et al. 1993). In inbred laboratory mice, susceptibility to infection originates from a single nonconservative glycine-to-aspartic acid substitution at position 169 within the transmembrane domain 4 of the *Slc11a1/Nramp1* gene (Malo et al. 1994, Vidal et al. 1996). Functionally, murine NRAMP proteins influence pathogen viability and/or replication within macrophages by transporting iron and other divalent cations across the phagosomal membrane (Gruenheid & Gros 2000).

The human homologue *Slc11a1/Nramp1* gene has been cloned and sequenced. This gene is located in the human chromosome region 2q35. It consists of 15 exons encoding a protein of 550 amino acids that exhibits 85% identity (92% similarity) with murine Nramp1 (Cellier et al. 1994, Blackwell et al. 1995). In addition, a microsatellite variant located in the immediate 5’ region of the gene, four variants in the coding region, three variants in introns, and one variant located in the 3’untranslated region (UTR) of the *Slc11a1/Nramp1* gene have been described, but the functional significance of these variants remains uncertain (Liu et al. 1995). Studies on the human homologue*Slc11a1/Nramp1* have linked/associated the region/gene with leprosy phenotypes (Abel et al. 1995, Meisner et al. 2001, Hatta et al. 2010). A meta-analysis study has confirmed the association of *Slc11a1/Nramp1* gene polymorphisms with susceptibility to tuberculosis (Li et al. 2011). In this regard, a population-based association study in Brazil has shown that lepromin-negative individuals harbouring specific genotypes of a (GT)n promoter repeat of the*Slc11a1/Nramp1* gene are at risk of developing leprosy, suggesting an interplay between genetic and immunological factors (Ferreira et al. 2004).

In the present case-control study, we have evaluated the association of nine polymorphisms [(GT)n, 274C/T, 469+14G/C, 577-18G/A, 823C/T, 1029 C/T, 1465-85G/A, 1703 G/A, and 1729+55del4] of the *Nramp1/Slc11a1* gene with susceptibility to leprosy or the clinical forms of leprosy.

## SUBJECTS, MATERIALS AND METHODS

*Subjects* - Eighty-six patients with leprosy from the leprosy outpatients unit of the University Hospital of the Ribeirão Preto Medical School, University of São Paulo, São Paulo, Brazil were recruited from 2002-2006. Leprosy classification was based on clinical and histological criteria (Ridley & Jopling 1966). Besides dermatological and neurological exams, lymph smear bacilloscopy, skin biopsy, Mitsuda test, and serology for anti-PGL1 were used.

Sixty-seven patients were classified as having MB leprosy [15 females (mean age = 38 ± 17 years) and 52 males (mean age = 49 ± 15 years)], which included nine, seven, 16, and 35 patients with the mid-BB, borderline-tuberculoid (BT), borderline-lepromatous, and LL clinical forms, respectively. Nineteen patients classified with PB leprosy [8 females (mean age = 45 ± 21 years) and 11 males (mean age = 49 ± 18 years)], which included 13 and six patients with the TT and BT clinical forms, respectively, were also included in this study. All the 239 healthy controls were blood donors affiliated with the Blood Bank of the University Hospital of Ribeirão Preto Medical School. The patients and controls were from the same region and were matched by age, gender, and skin colour. All the subjects provided an informed consent as required by the Institutional Ethical Committee of the University Hospital of Ribeirão Preto Medical School.

*Slc11a1/Nramp1 genotyping* - DNA was isolated from peripheral blood leukocytes by the salting-out method (Miller et al. 1988). A total of 100 ng of genomic DNA were amplified in 25 µL (final volume) of a reaction mixture containing 1x reaction buffer (20 mM Tris HCl pH 8.4, 50 mM KCl, 1.5 mM MgCl_2_, and 0.01% BSA), 10 mM dNTP, 2.5 mM of each primer, and 1.0 U Taq polymerase (Invitrogen, USA). The*Slc11a1/Nramp1* gene polymorphisms [(GT)n (rs34448891), 274C/T (rs2276631), 469+14G/C (rs3731865), 577-18G/A (rs3731864), 823C/T (rs17221959), 1029C/T (rs201565523), 1465-85G/A (rs2279015), 1703G/A (rs17235409), and 1729+55del4 (rs17235416)] were detected through polymerase chain reaction (PCR) followed by enzymatic restriction, according to protocols described by Liu et al. (1995), Singal et al. (2000), and Graham et al. (2000), with some modifications.

*Statistical analysis* - Genotype and allele frequencies were analysed by Fisher’s exact test. Logistic regression analyses were employed to assess the association between these polymorphisms and the disease. The Hardy-Weinberg equilibrium (HWE) was analysed by using the Genepop 4.2 software. The level of significance was set at p ≤ 0.05 in all the analyses. Data were analysed with the aid of the Statistical Analyses System software v.9.3 (SAS Institute, USA).

## RESULTS

Table I lists the distribution of the frequency of the (GT)n, 274C/T, and 469+14G/C *Slc11a1/Nramp1* gene polymorphisms in patients with leprosy and control subjects as well as the association tests of these polymorphisms for the different spectra of leprosy. Regarding the (GT)n polymorphism, the frequency of allele 2 in leprosy patients was higher as compared with the control group [32% vs. 24%; p = 0.04, odds ratio (OR) = 1.49, 95% confidence interval (CI) = 1.02-2.18]; the frequency of allele 3 was higher in the control group (74% vs. 65%; p = 0.03; OR = 0.66, 95% CI = 0.45-0.96). Analysis of the 274C/T polymorphism revealed increased frequency of the 274T allele (33% vs. 24%, p = 0.04; OR = 1.49, 95% CI = 1.02-2.18) and the T/T genotype (14% vs. 7%, p = 0.02; OR = 2.46, 95% CI = 1.08-5.62) among leprosy patients as compared with the control group. Similarly, a significantly higher frequency of the 469+14C allele of the 469+14G/C polymorphism occurred in leprosy patients as compared with the controls (30% vs. 23%, p = 0.03; OR = 1.53, 95% CI = 1.03-2.25). From a statistical viewpoint, the leprosy and control groups did not differ in terms of the genotypic and allelic frequencies of the *Slc11a1/Nramp1* 823C/T, 1465-85G/A, 1703 G/A, and 1729+55del4 polymorphisms (Table II). Regarding the 577-18G/A and 1029C/T polymorphisms, all the participants showed the genotypes “GG” and “CC”, respectively (data not shown). None of the nine polymorphisms of the *Slc11a1/Nramp1* gene was significantly different among the clinical forms of leprosy. The genotype distributions in the control group agreed with the HWE for all the polymorphisms.

**TABLE I:** Allelic and genotypic frequencies of the natural resistance-associated macrophage protein 1/solute carrier family 11 member 1 gene polymorphisms in leprosy and control groups

Polymorphism site	Alleles/ genotypes	Leprosy n = 86 n (%)	MB n = 67 n (%)	PB n = 19 n (%)	Controls n = 239 n (%)
Promoter microsatellite	Allele				
(GT)n repeat	1	1 (0.5)	1 (0.5)	0 (0)	4 (1)
	2	56 (32.5)	43 (32)	13 (34)	117 (24)^*a*^
	3	112 (65)	88 (66)	24 (63)	353 (74)^*b*^
	5	3 (2)	2 (1.5)	1 (3)	4 (1)
	Genotype				
	12	0 (0)	0 (0)	0 (0)	1 (0.5)
	13	1 (1)	1 (1.5)	0 (0)	3 (1)
	22	13 (15)	11 (16.5)	2 (11)	16 (7)
	23	28 (33)	20 (30)	8 (42)	83 (35)
	25	2 (2)	1 (1.5)	1 (5)	1 (0.5)
	33	41 (48)	33 (49)	8 (42)	132 (55)
	35	1 (1)	1 (1.5)	0 (0)	3 (1)
Exon 3	Allele				
274C/T	C	116 (67)	92 (68)	24 (63)	361 (76)
	T	56 (33)	42 (32)	14 (37)	117 (24)^*c*^
	Genotype				
	CC	42 (49)	34 (51)	8 (42)	138 (58)
	CT	32 (37)	24 (36)	8 (42)	85 (35)
	TT	12 (14)	9 (13)	3 (16)	16 (7)^*d*^
Intron 4	Allele				
469+14 G/C	C	53 (30)	40 (30)	13 (34)	108 (23)^*e*^
	G	119 (70)	94 (70)	25 (66)	370 (77)
	Genotype				
	CC	10 (12)	8 (12)	2 (11)	14 (6)
	CG	33 (38)	24 (36)	9 (47)	80 (33)
	GG	43 (50)	35 (52)	8 (42)	145 (61)

*a*: allele 2 [p = 0.04, odds ratio (OR) = 1.49, 95% confidence interval (CI) = 1.02-2.18]; *b*: allele 3 (p = 0.03; OR = 0.66, 95% CI = 0.45-0.96); *c*: 274T allele (p = 0.04; OR = 1.49, 95% CI = 1.02-2.18);*d*: TT genotype (p = 0.02; OR = 2.46, 95% CI = 1.08-5.62); *e*: 469+14C allele (p = 0.03; OR = 1.53, 95% CI = 1.03-2.25); MB: multibacillary; PB: paucibacillary.

**TABLE II:** Allelic and genotypic frequencies of the natural resistance-associated macrophage protein 1/solute carrier family 11 member 1 gene polymorphisms in leprosy and control groups

Polymorphism site	Alleles/ genotypes	Leprosy n = 86 n (%)	MB n = 67 n (%)	PB n = 19 n (%)	Controls n = 239 n (%)
Exon 8	Allele				
823 C/T	C	152 (88)	119 (88)	33 (86)	430 (89)
	T	20 (12)	15 (12)	5 (14)	48 (11)
	Genotype				
	CC	69 (80)	54 (81)	15 (79)	195 (81)
	CT	14 (16)	11 (16)	3 (16)	40 (17)
	TT	3 (4)	2 (3)	1 (5)	4 (2)
Intron 13	Allele				
1465-85 G/A	G	81 (47)	63 (47)	18 (47)	256 (54)
	A	91 (53)	71 (53)	20 (53)	222 (46)
	Genotype				
	GG	22 (26)	16 (24)	6 (31)	79 (33)
	GA	37 (43)	31 (46)	6 (31)	98 (41)
	AA	27 (31)	20 (30)	7 (38)	62 (26)
Exon 15	Allele				
1703 G/A	G	168 (97)	131 (98)	37 (99)	462 (96)
	A	4 (3)	3 (2)	1 (1)	16 (4)
	Genotype				
	GA	4 (5)	3 (4)	1 (5)	16 (7)
	GG	82 (95)	64 (96)	18 (95)	223 (93)
3’ UTR	Allele				
1729+55del4	+TGTG	161 (94)	125 (93)	36 (95)	440 (92)
	-TGTG	11 (6)	9 (7)	2 (5)	38 (8)
	Genotype				
	+TGTG	76 (88)	59 (88)	17 (89)	204 (85)
	+TGTG/-TGTG	9 (10)	7 (10)	2 (11)	32 (13)
	-TGTG	1 (2)	1 (2)	0 (0)	3 (2)

MB: multibacillary; PB: paucibacillary; UTR: untranslated region.

## DISCUSSION

Positive and negative associations of the *Nramp1/Slc11a1* gene polymorphisms with leprosy have been described (Roy et al. 1999, Fitness et al. 2004,Hatta et al. 2010).

This case-control study revealed that the promoter microsatellite (GT)n and the 274C/T and 469+14G/C polymorphisms of the *Slc11a1/Nramp1* gene are associated with leprosy.

The microsatellite (GT)n repeat in the promoter region of the*Slc11a1/Nramp1* gene is functional during transcription regulation (Blackwell et al. 1995, Searle & Blackwell 1999, Bayele et al. 2007). Nine variants have been described at this site (Blackwell et al. 1995,Graham et al. 2000, Kojima et al. 2001, Zaahl et al. 2004). In reporter gene studies, Searle and Blackwell (1999) have shown that these four alleles differ in their ability to drive gene expression. Allele 3 at the GTn polymorphism drives higher expression of the *Slc11a1/Nramp1* gene, whilst allele 2 drives lower *Slc11a1/Nramp1* expression in the presence of bacterial lipopolysaccharide. These authors suggested that the higher expression of allele 3 should drive pro-inflammatory activated macrophage responses that are functionally linked to susceptibility to autoimmune disease. On the other hand, the lower level of *Slc11a1/Nramp1* expression promoted by allele 2 should contribute to susceptibility to infectious disease (Searle & Blackwell 1999, Blackwell et al. 2003). In our study, we have observed that the frequency of allele 2 of the promoter microsatellite (GT)n of the *Slc11a1/Nramp1* gene is higher in the leprosy group, which suggests its association with susceptibility to leprosy. In line with our results, Ferreira et al. (2004) verified that individuals with a negative lepromin response associated with genotypes “22” and “23” presented greater risk of developing leprosy. In contrast to our study, a candidate gene study in a population from northern Malawi (Fitness et al. 2004)and India (Roy et al. 1999) showed no association of the microsatellite (GT)n repeat in the promoter region of the*Slc11a1/Nramp1* gene with leprosy.

Here, we have found that two other single nucleotide changes in exon 3 (274C/T) and intron 4 (469+14G/C) of the *Slc11a1/Nramp1* gene are associated with susceptibility to leprosy. Leprosy patients carry the 274T allele and TT homozygosis of the 274C/T polymorphism and the 469+14C allele of the 469+14G/C polymorphism more frequently than the controls. These results may help to prevent the leprosy disease by screening for the 274C/T and 469+14G/C polymorphisms in healthy contacts. To our knowledge, there are few reports about the 274C/T variation of the*Slc11a1/Nramp1* gene in leprosy. Recently, a Brazilian study of 201 leprosy cases revealed that the presence of the homozygosis mutant “TT” was related to risk for type 2 reaction (Teixeira et al. 2010). Regarding the 469+14G/C polymorphisms, Hatta et al. (2010) evaluated three variants of the*Nramp1/Slc11a1* gene (1703G/A, 1729+55del4, and 469+14G/C) and found that the “GC” heterozygosis of the 469+14G/C polymorphism was associated with leprosy *per se* and with PB leprosy in patients from Indonesia. In contrast to our result, several reports have not detected any association of the 469+14G/C polymorphism with leprosy (Roy et al. 1999, Meisner et al. 2001, Vejbaesya et al. 2007). The physiological effect of these polymorphisms of the *Nramp1/Slc11a1* gene is not fully understood.

We have also evaluated six (577-18G/A, 823C/T, 1029C/T, 1465-85G/A, 1703G/A, and 1729+55del4) other *Slc11a1/Nramp1* gene polymorphisms, but we have not found any association with leprosy. In a case-control study in Mali, the 3’ UTR (1729+55del4) polymorphisms were specifically associated with MB leprosy, but not with leprosy *per se* (Meisner et al. 2001). However, candidate gene studies in a population from northern Malawi (Fitness et al. 2004), India (Roy et al. 1999), and Thailand (Vejbaesya et al. 2007) failed to detect any association between *Nramp1/Slc11a1* polymorphisms and leprosy.

Discrepancies exist in the polymorphisms of the *Slc11a1/Nramp1* gene associated with leprosy, and the functional significance of these polymorphisms remains unclear. In the mouse model, the *Slc11a1/Nramp1* alleles influence the intrinsic ability of macrophages to resist infection by intracellular parasites (Skamene 1994). Other studies have shown that the *Slc11a1/Nramp1 locus* encodes a protein that also exerts pleiotropic effects, including regulation of the Th1/Th2 balance of the adaptive immune response to infection (Soo et al. 1998, Blackwell et al. 2000). It is very important to identify the genetic factors that regulate the Th1/Th2 balance in response to foreign antigens. The polymorphisms of the *Slc11a1/Nramp1*gene could play an important role in the immune response against *M. leprae*.

In conclusion, our results have demonstrated that the 274C/T polymorphism in exon 3 and the 469+14G/C polymorphism in intron 4 of the *Slc11a1/Nramp1*gene are associated with susceptibility to leprosy. Furthermore, the (GT)n polymorphisms in the promoter region of the *Slc11a1/Nramp1* gene are associated with protection or susceptibility to leprosy.
